# In vitro performance of the Vortex Intelligent Stone Optimized Removal (VISOR) system versus the flexible and navigable suction ureteral access sheath (FANS) on stone clearance efficiency and intrarenal pressure control

**DOI:** 10.1007/s00345-026-06778-3

**Published:** 2026-09-17

**Authors:** Wenjun Gao, Li Fang, Jia-Lun Kwok, Baiyang Song, Steffi Kar Kei Yuen, Vineet Gauhar, Yue Cheng, Bohan Wang

**Affiliations:** 1 Department of Urology, The Second Affiliated Hospital, School of Medicine, Zhejiang University, Hangzhou, 310009 Zhejiang China; 2 Department of Urology, The First Affiliated Hospital of Ningbo University, 59 Liuting Street, Haishu District, Ningbo, 315000 Zhejiang P.R. China; 3 Zhejiang Engineering Research Center of Innovative Technologies and Diagnostic and Therapeutic Equipment for Urinary System Diseases, Hangzhou, Zhejiang China; 4 Department of Urology, Tan Tock Seng Hospital, Singapore, Singapore; 5 Lee Kong Chian School of Medicine, Nanyang Technological University, Singapore, Singapore; 6 S.H. Ho Urology Centre, Department of Surgery, The Chinese University of Hong Kong, Hong Kong, China; 7 Department of Urology, Ng Teng Fong Hospital, Singapore, Singapore; 8 Asian Institute of Nephrology and Urology, Hyderabad, India

**Keywords:** Nephrolithiasis, VISOR system, Intrarenal pressure, Stone clearance efficiency, In vitro model

## Abstract

**Introduction:**

Flexible and navigable suction ureteral access sheath (FANS) ureteroscopy has been recognized as an important development in the suction era of endourology. The Vortex Intelligent Stone Optimized Removal (VISOR) system has recently been introduced as an alternative to FANS, featuring an integrated irrigation-suction dual-channel and IRP control design. This study aimed to evaluate the efficacy of VISOR system in comparison with FANS in vitro.

**Methods:**

3D-printed renal models were used, and 3 g of human calcium phosphate stones were treated via VISOR system versus FANS ureteroscopy. All procedures were performed by a single experienced urologist with a holmium laser. Primary outcome was stone clearance efficiency (SCE), calculated as: SCE (%) = [(Initial stone mass − Residual stone mass) / Initial stone mass] × 100%. Secondary outcomes included operative time, total laser energy use, residual stone mass and total stone mass removed. Accuracy of IRP measurements was also tested for the VISOR system.

**Results:**

The VISOR system demonstrated comparable SCE to FANS (95.1 ± 0.6% vs. 94.8 ± 0.7%, *p* = 0.78). The mean operative time in the VISOR group was significantly lower by 26.2% compared with FANS (55.1 ± 2.2 vs. 74.7 ± 2.3 min, *p* = 0.003). We found no difference in total laser energy use (33.6 ± 1.4 vs. 33.3 ± 5.3 kJ, *p* = 0.96), residual stone mass (0.15 ± 0.03 vs. 0.15 ± 0.04 g, *p* = 0.99) and total removed stone mass (1.86 ± 0.13 vs. 1.81 ± 0.12 g, *p* = 0.79). The VISOR system’s pressure sensor showed good correlation with an external reference pressure meter (Spearman *R* = 0.99, *p* < 0.0001).

**Conclusions:**

Based on these in vitro findings, the VISOR integrated dual-channel system demonstrated SCE comparable to FANS, with significantly shorter operative time and reliable real-time IRP control. These results suggest that the VISOR system represents a promising candidate for further clinical evaluation; whether these performance advantages translate to improved patient outcomes in vivo requires confirmation in prospective clinical studies.

**Supplementary Information:**

The online version contains supplementary material available at https://doi.org/10.1007/s00345-026-06778-3.

## Introduction

Kidney stone disease (KSD) is a global health issue, with a lifetime prevalence of 5–10% and a 5-year recurrence rate of approximately 50% [1, 2]. Retrograde intrarenal surgery (RIRS) is a well-established first-line intervention for renal stones up to 2 cm, providing effective stone clearance with low morbidity [3]. Recently, the European Association of Urology (EAU) identified optimal control of suction, irrigation, intrarenal temperature and intrarenal pressure (IRP) as a crucial quadrifecta for maximizing zero residual fragment (ZRF) rates and minimizing infectious complications and sepsis [4].

Flexible and navigable suction ureteral access sheaths (FANS) have emerged to meet these goals, enabling active fragment aspiration and achieving non-inferior clinical outcomes akin to mini-PCNL [5–9]. However, significant challenges remain. Current FANS procedures require repeated scope withdrawal for fragment extraction, which increases surgeon fatigue and prolongs operative time, especially in large stone burdens [10]. Furthermore, integrating FANS with separate IRP monitors and fluid management systems increases capital costs, technical complexity, and the cognitive load on the surgeon [11, 12]. While alternative aspiration systems like CVAC^®^ show promise for large stones, they lack integrated IRP and fluid regulation [13]. Therefore, an “all-in-one” suction-scope system is highly desirable to make RIRS a seamless and ergonomically efficient procedure.

**Fig. 1:**
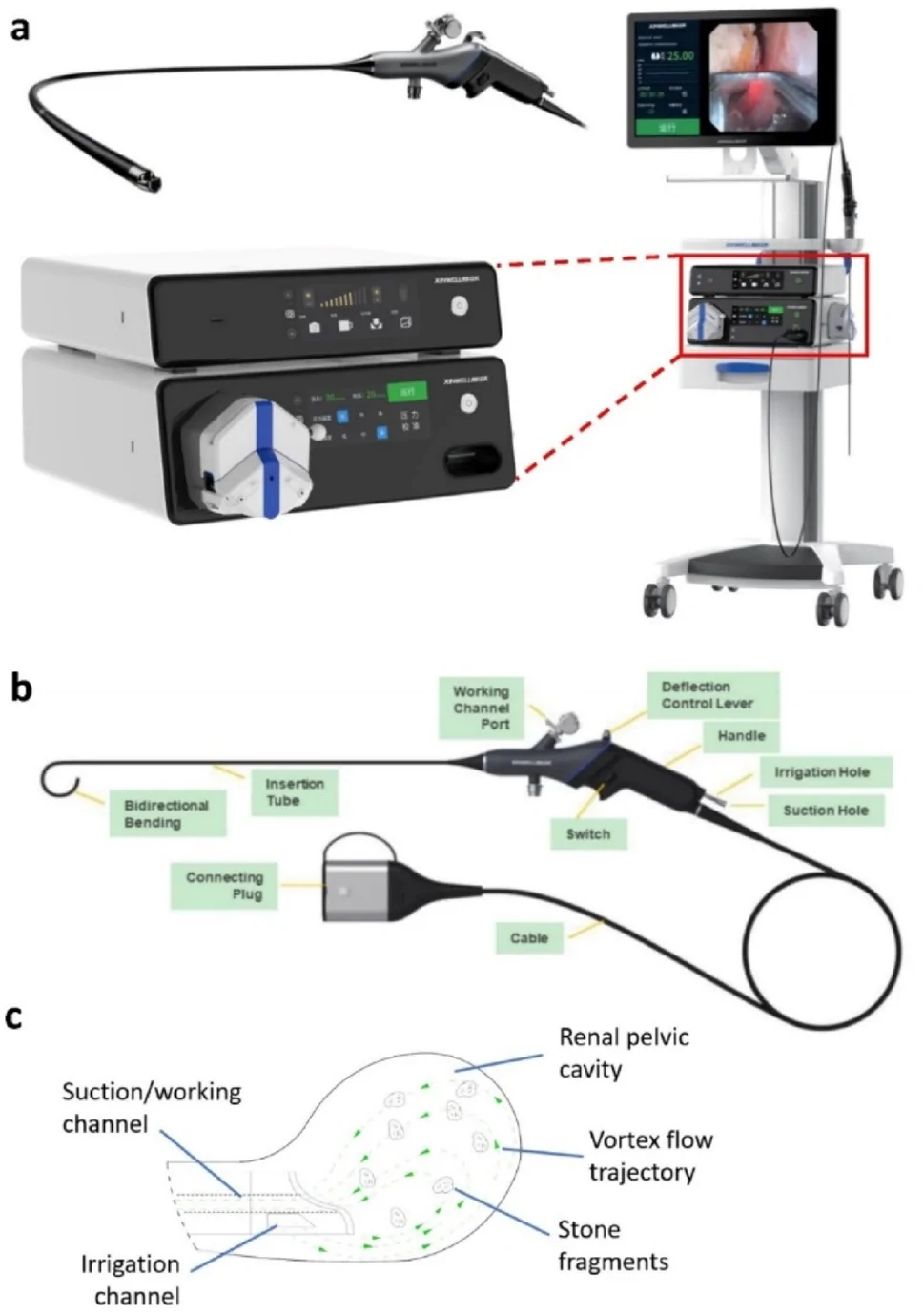
Components of the Vortex Intelligent Stone Optimized Removal (VISOR) system

Figure 1 describes the various parts of The Vortex Intelligent Stone Optimized Removal (VISOR, Xinwell Medical Technology, China) system, a novel integrated ureteroscopic platform that combines a dual-channel flexible ureteroscope for simultaneous irrigation and suction, with an artificial intelligence-guided irrigation-suction console that provides real-time IRP monitoring and automatic regulation of same during laser lithotripsy. Its proprietary in-built suction and evacuation of stone fragments scope tip may reduce the need for repeated scope withdrawal, improving the possibility of a single-session stone clearance efficiency (SCE) in ergonomically feasible way. The ability for automated fluid regulation of IRP can potentially minimize pyelovenous backflow, bacterial translocation, and urosepsis [14, 15]. Thus, all these features may address certain shortcomings of FANS and warrant further clinical investigation.

To evaluate its potential, we conducted an in vitro study using a 3D-printed renal model to compare the performance of the VISOR system against the mainstream FANS approach. The primary aim was to compare SCE, operative time, total laser energy use, and removed stone mass. The secondary aim was to validate the accuracy of the VISOR system’s integrated IRP measurements.

**a** Major components of the VISOR system, including a dual-channel flexible ureteroscope, a display monitor, an image console and an irrigation-suction console. **b** Photograph of the VISOR dual-channel flexible ureteroscope, highlighting the handle, working channel ports, irrigation/suction interface, and bidirectional bending insertion tube. **c** Schematic diagram illustrating the vortex capture mechanism within the renal pelvis: coordinated irrigation flow generates a recirculating vortex field that mobilizes stone fragments toward the suction channel inlet, facilitating continuous evacuation of sub-threshold particles (≤ 1.8 mm) during active lithotripsy.

## Materials and methods

### 3D-printed renal model

**Fig. 2:**
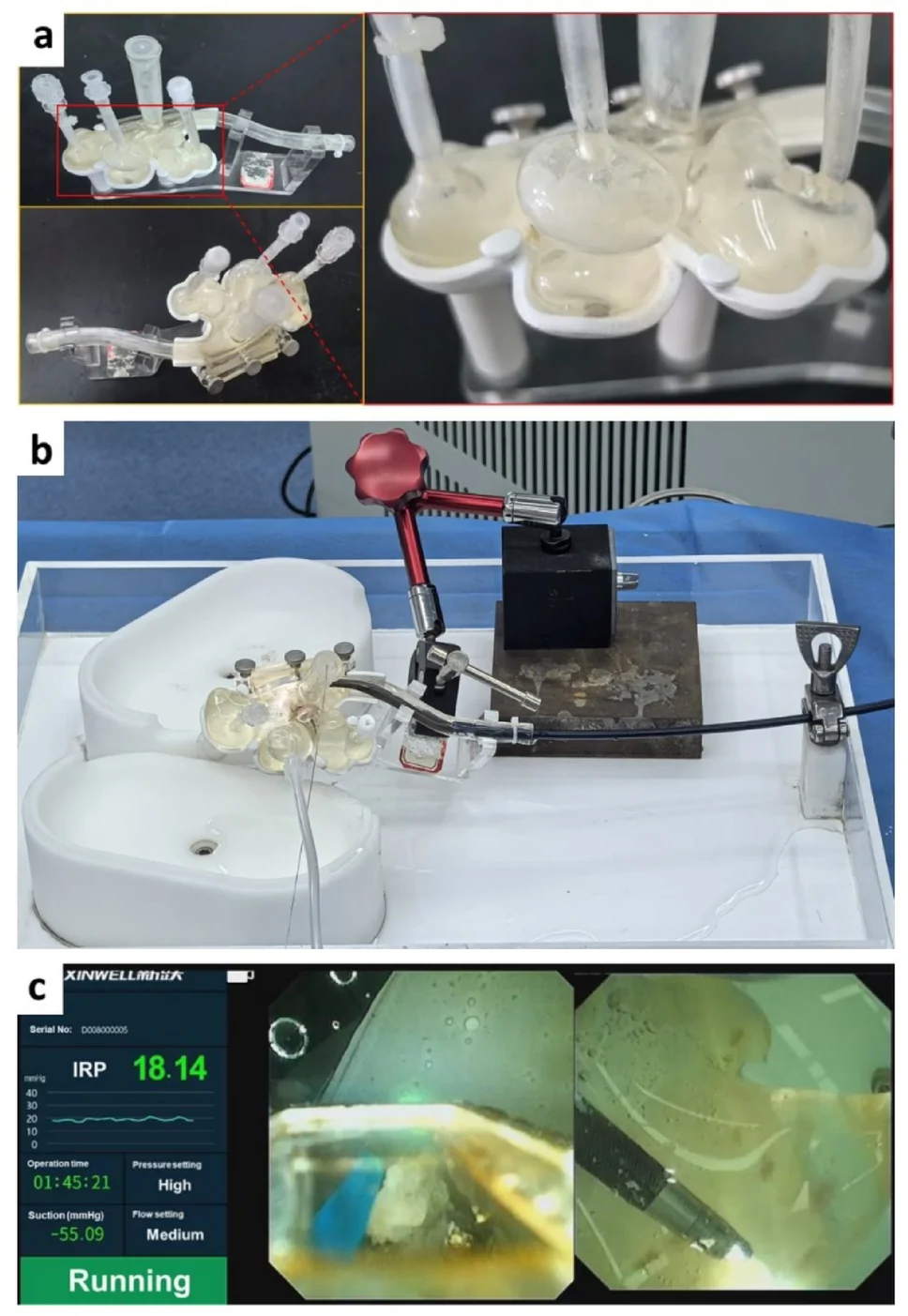
3D-printed renal model and experimental setup

An anatomically accurate 3D-printed renal model was fabricated from medical-grade silicone (elastic modulus: 0.5 MPa) using clinical computed tomography urography (CTU) data of an adult patient with normal upper urinary tract anatomy per previously described methods [16]. The model fully replicated the structure of the renal pelvis, major and minor calyces, and ureter. Multiple dedicated pressure-monitoring channels were embedded at the lateral wall of the renal pelvis and calyces for real-time IRP recording via COMET™ II pressure guidewire (Boston Scientific, Marlborough, MA, USA). The model was fixed in a simulated anatomical position during all procedures to ensure consistent experimental conditions (Fig. 2b).

### Stone preparation

Human urinary stones were obtained from consenting patients who underwent open cystolithotomy in our institution. The stones were washed with distilled water, dried in a drying oven at 60 °C until the weight remained unchanged, and processed into standardized cuboids (1.6 × 1.0 × 1.0 cm, mass ≈ 3 g) using a precision cutting machine. Infrared spectroscopy confirmed a mixed composition with calcium phosphate as the predominant component (> 90%). Each stone fragment was weighed using an analytical balance with a precision of 0.001 g (Mettler Toledo, Zurich, Switzerland) before the experiments.

### Study groups and experimental devices

This in vitro study included two parallel groups: the VISOR group (integrated dual-channel ureteroscope system) and the FANS group (single-channel ureteroscope with suction ureteral access sheath), with 3 independent replicates per group (*n* = 3 per group).

### Experimental protocol

All procedures were performed by a single urologist to eliminate operator-related bias. For each replicate, the standardized 3 g stone was placed in the renal pelvis of the 3D-printed model. After insertion of the UAS, the open end of the proximal ureter of the model was sealed with a self-tightening tie to minimize fluid leakage and enhance the reliability of IRP detection (Fig. 2a, b). A standardized holmium: YAG laser (Lumenis, Israel) setting of 1 J × 30 Hz (30 W) [17] was used across both groups to isolate the effect of suction technology on stone clearance outcomes.

#### Procedure for the VISOR group

#####  Description of the VISOR technology

The VISOR system (Xinwell Medical Technology, China) consists of a 12 Fr dual-channel flexible ureteroscope (3.3 Fr irrigation channel, 5.4 Fr dedicated suction/working channel), an image processing console, and a proprietary automated irrigation-suction console with intelligent integrated real-time IRP monitoring (Fig. 1). It was paired with a 13/14 Fr non-suction ureteral access sheath (UAS, Xinwell Medical Technology, China) for ureteral access, emulating clinical practice. The system was preset with four-tiered safety thresholds: (1) an audio-visual IRP alarm at 35 mmHg; (2) an automatic irrigation shutdown at an IRP of 40 mmHg; (3) an audio-visual suction circuit alarm at − 280 mmHg for early detection of potential channel occlusion; (4) automatic irrigation shutdown triggered by suction circuit pressure at − 350 mmHg to mitigate potential channel occlusion. Suction channel patency was assessed throughout each replicate by continuous monitoring of the suction circuit vacuum pressure. The irrigation flow rate was set to 155 ml/min by the irrigation-suction console by default.

##### VISOR procedure

The VISOR dual-channel ureteroscope was inserted into the renal pelvis through the 13/14 Fr UAS, and the vortex irrigation-suction mode (preset in the system) was activated during lithotripsy process. Stone fragments smaller than 1.8 mm were simultaneously and continuously aspirated through the dedicated 5.4Fr suction channel of the ureteroscope (Fig. 2c). The scope was not required to be withdrawn throughout the procedure; only fragments ≤ 1.8 mm were evacuated through the dedicated 5.4Fr suction channel, while continuous laser fragmentation was maintained until the effective stone burden was cleared. IRP was continuously monitored, both by the system’s built-in distal-tip pressure sensor and the external pressure meter, which acted as a control to verify IRP measurements with a raw sampling frequency of 1 Hz (1 data record per second).

#### Procedure for the FANS group

##### FANS technical considerations

Procedures were performed with a 7.5-Fr single-use digital flexible ureteroscope (ZebraScope™, Happiness Works Medical Instruments Co., Ltd, Anhui, China) paired with a 12/14 Fr FANS (ELEPHANT-Ⅱ, Zhejiang YiGao Medical Technology, Hangzhou, China). The FANS-UAS was connected to an external negative pressure suction device, with the negative pressure set to 0.02 MPa. The irrigation flow rate was set to 100 ml/min using a standard irrigation system (ISA-IIIa, Hawk, Shenzhen, China), reflecting standard clinical practice [8].

##### FANS procedure

The 12/14 Fr FANS was placed into the renal pelvis, followed by insertion of the 7.5-Fr flexible ureteroscope. Laser lithotripsy was performed in a conventional manner, using the same laser setting as the VISOR group. Periodically, the ureteroscope was withdrawn from the FANS to enable fragment aspiration through the sheath as has been described [18].

**a** 3D-printed silicone renal model, replicating the renal pelvis, calyces, and ureter of a normal adult upper urinary tract. Left panels: different angles of the complete model. Right panel: enlarged view of the renal pelvis. **b** Overall experimental setup, including the fixed renal model, ureteral access sheath (UAS) and ureteroscope insertion, external pressure meter for intrarenal pressure (IRP) monitoring (for VISOR group only), and supporting equipment. After insertion of the ureteral access sheath, the open end of the proximal ureter was sealed with a self-tightening tie to minimize fluid leakage. **c** The left and middle panels represent the display interfaces of the VISOR system. The left interface shows intraoperative surgery-related data, and the middle panel shows real-time lithotripsy. The right panel displays the surgical procedure recorded from outside the 3D-printed renal model.

### Outcome measures

Primary outcome was stone SCE, calculated as: SCE (%) = [(Initial stone mass - Residual stone mass)/Initial stone mass] × 100%.

Secondary outcomes included (1) operative time (min), defined as the time from first ureteroscope insertion to the completion of lithotripsy and fragment clearance; (2) Total removed stone mass (g), defined as the dry weight of discrete stone fragments recovered from the sealed suction collection bottle, plus any fragments retrieved by dissecting and flushing the renal model; This measure does not capture very fine particles that passed through the filter mesh and remained suspended in the irrigation effluent; such fine debris accounted for the discrepancy between total removed mass and the initial stone load.; and (3) total laser energy use (J) during lithotripsy. Lastly, IRP control performance between the VISOR system’s built-in sensor and the external reference pressure meter was measured. After each procedure, the 3D-printed renal model was dissected. Any residue was fully retrieved by repeated flushing with normal saline, and the renal pelvis cavity inspected under a stereomicroscope to ensure no residual material was missed. All aspirated stone fragments were separately collected from the sealed stone collection bottle of each group. Both residual and cleared stone fragments were dried in a constant-temperature drying oven at 60 °C until the weight remained unchanged and weighed using the same analytical balance.

### Statistical analysis

For data analysis and visualization, we down-sampled the raw 1 Hz dataset to a 6-second interval (1 data point every 6 s). All statistical analyses (including Bland-Altman and Spearman correlation) and figure plotting were performed based on the down-sampled dataset with 6-second intervals.

Normality of continuous data was verified using the Shapiro-Wilk test. For operative time, SCE, laser energy, and stone mass (*n* = 3 per group), normally distributed data are presented as mean ± standard error of the mean (SEM), and intergroup differences were analyzed using the independent samples Student’s t-test. For intraoperative IRP data, which demonstrated non-normal distribution due to the presence of transient pressure spikes, data are presented as median (interquartile range, IQR) with minimum and maximum values. The agreement between the two IRP measurement methods was evaluated using Spearman correlation analysis and Bland-Altman plots. All statistical analyses were performed using GraphPad Prism 10.1.2 (GraphPad Software, San Diego, CA, USA). A two-sided *p* < 0.05 was considered statistically significant.

## Results

### Baseline stone burden

**Fig. 3:**
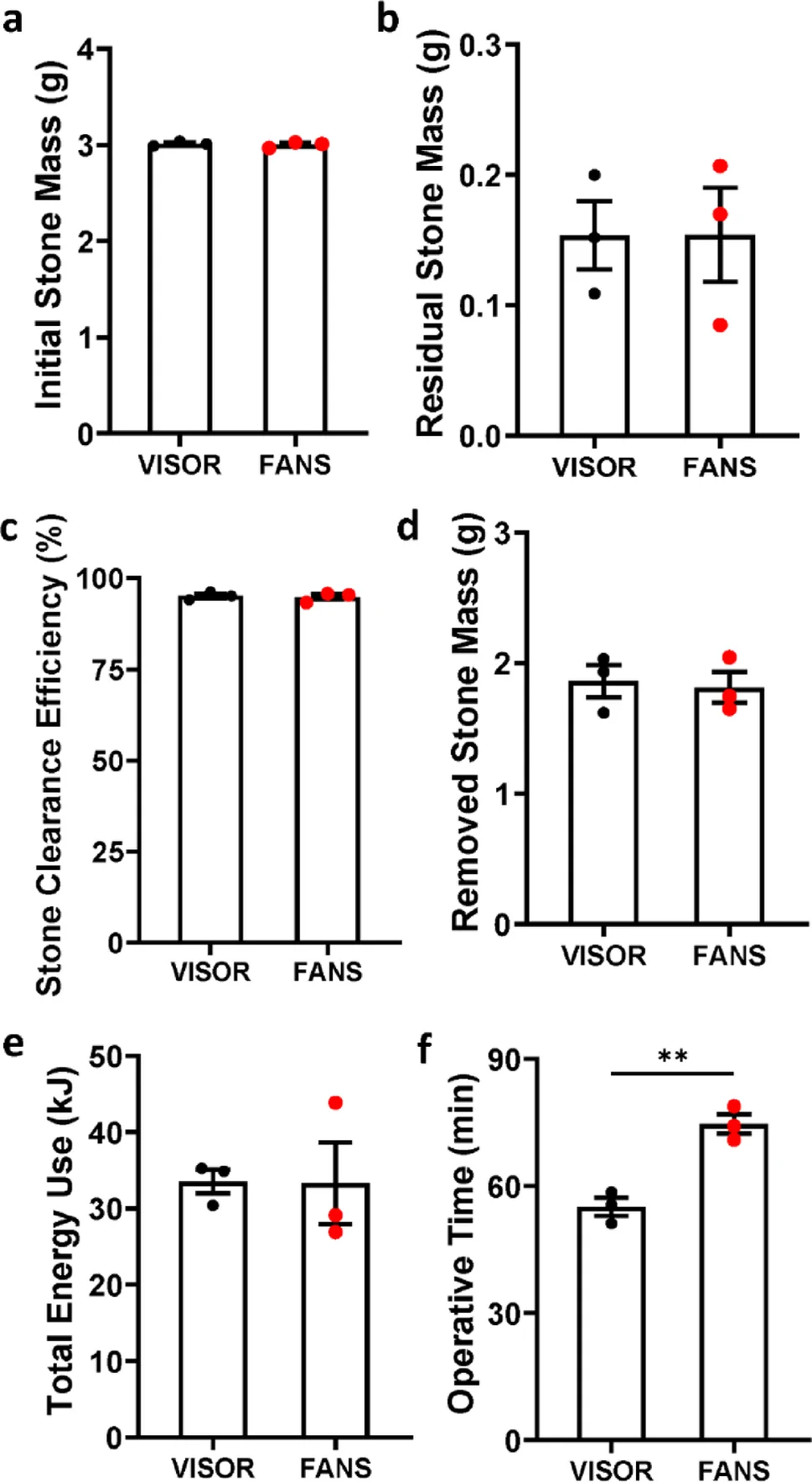
Comparative outcomes between the VISOR and FANS groups

Initial stone mass was similar between the VISOR and FANS groups (3.01 ± 0.01 g vs. 3.00 ± 0.02 g, respectively; *p* = 0.64; Fig. 3a).

### Primary outcome

The VISOR system demonstrated comparable SCE compared with FANS, achieving 95.1 ± 0.6% versus 94.8 ± 0.7%, respectively (*p* = 0.78; Fig. 3c).

### Secondary outcomes

Residual stone mass was comparable between the VISOR and FANS groups (0.15 ± 0.03 g vs. 0.15 ± 0.04 g, respectively; *p* = 0.99; Fig. 3b). Total removed stone mass also did not differ significantly between groups (1.86 ± 0.13 g vs. 1.81 ± 0.12 g; *p* = 0.79; Fig. 3d), and total laser energy use was similar (33.6 ± 1.4 kJ vs. 33.3 ± 5.3 kJ; *p* = 0.96; Fig. 3e). In contrast, a 26.2% reduction in mean operative time was noted in the VISOR group compared with the FANS group (55.1 ± 2.2 min vs. 74.7 ± 2.3 min; *p* = 0.003; Fig. 3f). The sum of recovered removed mass and residual mass across all replicates accounted for approximately 2.0 g of the initial ~ 3 g stone load. The unrecovered ~ 1 g fraction represents very fine particles and near-dust debris that were suspended in the irrigation effluent and passed through the stone collection bottle filter.

**a** Initial mass of stones placed in the renal pelvis before lithotripsy. **b** Residual stone mass remaining in the renal model after the procedure. **c** Stone clearance efficiency (SCE) of the two groups. **d** Total removed stone mass during the procedure. **e** Total laser energy use during lithotripsy. **f** Mean operative time of the two groups. Data are presented as mean ± standard error of the mean (SEM). Individual data points are plotted over bar charts showing mean ± SEM (*n* = 3 per group). Black dots, VISOR; red dots, FANS. ** *p* < 0.01 vs. VISOR group. Individual dots represent measurements from individual experimental runs. VISOR: vortex intelligent stone optimized removal system; FANS, flexible and navigable suction ureteral access sheath (Table 1).

**Table 1:** Summary of comparative outcomes between the VISOR and FANS groups

Parameter	VISOR (*n* = 3)	FANS (*n* = 3)	*p*-value
Initial stone mass (g)	3.01 ± 0.01	3.00 ± 0.02	0.64
Residual stone mass (g)	0.15 ± 0.03	0.15 ± 0.04	0.99
Stone clearance efficiency (%)	95.1 ± 0.6	94.8 ± 0.7	0.78
Removed stone mass (g)	1.86 ± 0.13	1.81 ± 0.12	0.79
Operative time (min)	55.1 ± 2.2	74.7 ± 2.3	0.003
Total energy use (kJ)	33.6 ± 1.4	33.3 ± 5.3	0.96
Median IRP — VISOR (mmHg)	16.49 (IQR 14.71–19.79)	N/A	—
Maximum IRP — VISOR (mmHg)	37.91	N/A	—

Data are presented as mean ± SEM unless otherwise specified. Median IRP is reported as median (IQR: interquartile range) as this parameter is based on continuous intraoperative time-series measurements rather than three discrete experimental replicates. Maximum IRP represents the single highest recorded value across all VISOR replicates. IRP was measured only in the VISOR group; N/A, not applicable. Statistical comparisons were performed using unpaired two-tailed Student’s t-test (*n* = 3 per group). IQR, interquartile range; IRP, intrarenal pressure.

### IRP control performance

**Fig. 4:**
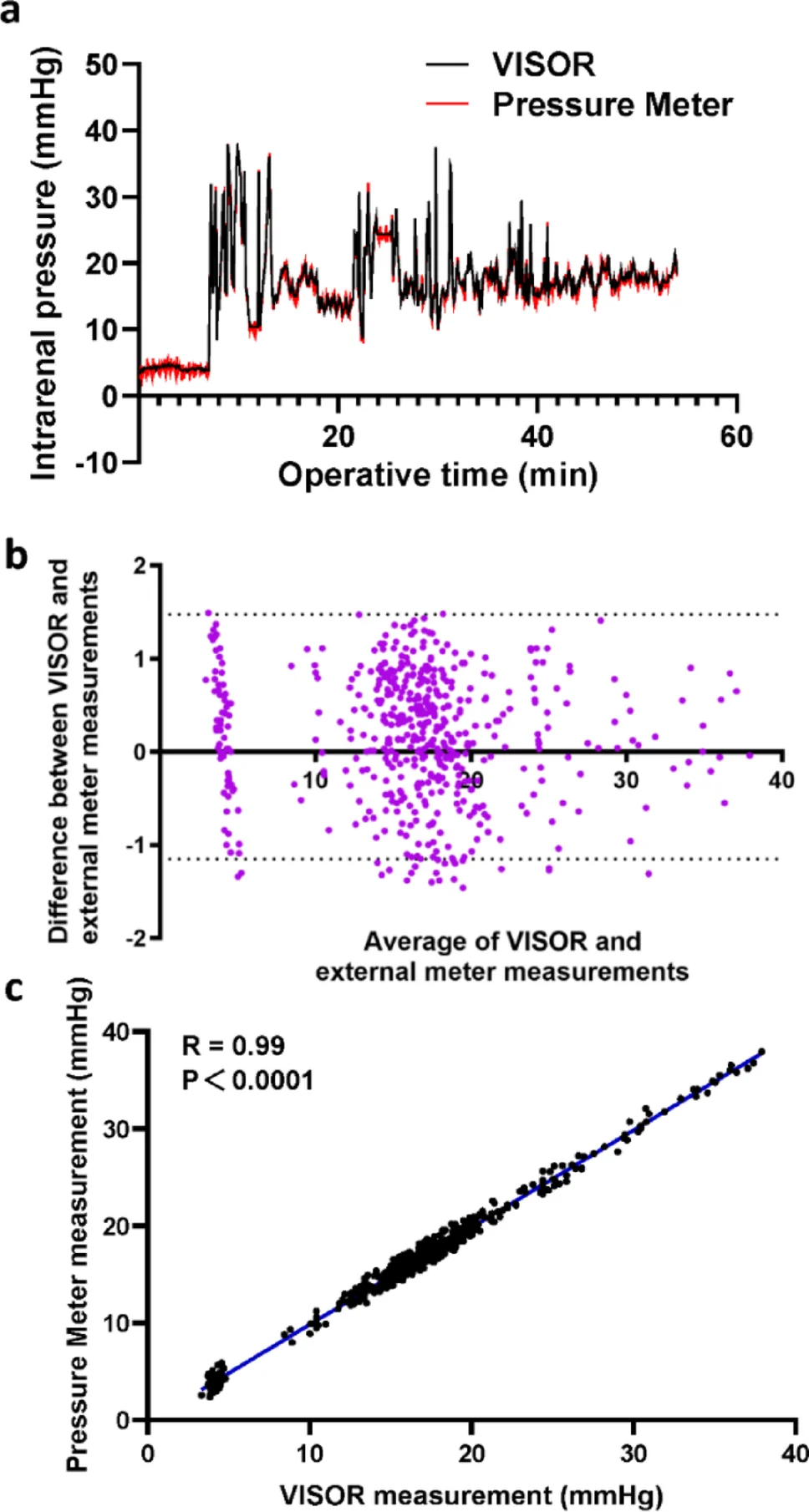
Comparison of mean intrarenal pressure (IRP) measured by the VISOR system versus the external pressure meter

During VISOR procedures, the median IRP was 16.49 mmHg (IQR 14.71–19.79 mmHg), with a maximum recorded pressure of 37.91 mmHg. IRP trends recorded by the VISOR system’s built-in sensor closely matched those measured by the external pressure meter (Fig. 4a). Bland–Altman analysis showed a mean bias of 0.16 mmHg between methods, with 95% limits of agreement from − 1.15 to 1.47 mmHg, indicating strong agreement (Fig. 4b). Spearman correlation analysis further demonstrated a significant positive correlation between the two methods (*R* = 0.99, *p* < 0.0001; Fig. 4c).

**a** IRP values recorded every 6 s by the VISOR system’s built-in pressure sensor and the external reference pressure meter. **b** Bland–Altman plot of the IRP measurements from VISOR system’s built-in pressure sensor and the external pressure meter. **c** Spearman correlation analysis of IRP measurements between the VISOR system and the external pressure meter (*R* = 0.99, *p* < 0.0001). The plot used in Fig. 4 was generated based on the preprocessed dataset with a 6-second sampling interval from the original 1 Hz continuous monitoring data.

### Suction channel patency

No suction channel obstruction events were observed across all three VISOR experimental replicates. The 5.4 Fr suction channel remained patent throughout all procedures without requiring scope withdrawal, manual flushing, or procedural interruption. The integrated negative-pressure monitoring system was designed to detect channel occlusion via an increase in the magnitude of negative pressure within the suction circuit, with an audio-visual alarm triggered at − 280 mmHg and automatic irrigation shutdown activated at − 350 mmHg as a tiered protective response; neither the alarm nor the shutdown mechanism was triggered during the study. In contrast, the FANS group inherently required intermittent scope withdrawal to allow fragment evacuation through the access sheath.

## Discussion

### Clinical context and rationale for integrated systems

FANS has been validated in multiple clinical studies as an effective approach for renal stone clearance and its ability to achieve a zero residual fragment status even in children and the elderly, and is comparable to standard PCNL and even suction mini-PCNL [6, 8, 9, 19–23], and has been described as a meaningful advancement in the endoscopic management of KSD.

However, FANS requires repeated withdrawal of the ureteroscope to allow relatively large fragments to be aspirated through the sheath. This process interrupts lithotripsy and can become ergonomically tedious, peri-operatively tiring for larger stone burdens with repeated scope pull outs, thus potentially prolonging operative time [8, 24]. Further, whilst FANS does help reduce IRP and clinical studies do report benefits of safer, faster and better outcomes using FANS with IRP control [25–28], current applications often require either specialized scopes or accessory IRP recording devices, necessitating additional suction irrigation pressure autoregulating systems (SIPS) [29], which increase capital cost and complexity, and may best suit only experienced or high-volume centers. Yet, these considerations of proper fluid management, automated IRP management and efficient suction in flexible ureteroscopy suggest a rationale for investigating fully integrated all-in-one system like VISOR.

### Operative efficiency and ergonomics

In our experiment, the VISOR group had a significantly shorter mean operative time (*p* = 0.003). Two mechanisms may explain this improvement. First, the VISOR system’s proprietary tip design (Fig. 1c) and intelligent suction-irrigation console (patent held by Xinwell Medical Technology, China), creates a stable vortex effect exactly at the scope tip, actively drawing stone fragments toward the laser fiber for continuous fragmentation. This is fundamentally different from the sheath-based aspiration of FANS, which relies on a pressure gradient generated within the peri-scope annular space. The efficiency of this annular clearance highly depends on the delicate balance between irrigation inflow and external suction, which can be variable depending of the sheath size used, the PCS capacity, and the presence or absence of hydronephrosis [30, 31]. Second, the VISOR’s dual-channel design enables simultaneous active automated fragment aspiration without repeated scope withdrawal unlike that of FANS. This in theory has the potential benefit of reducing surgeon fatigue. Furthermore, avoiding repeated scope withdrawal may reduce the potential for accidental injury to the PCS.

From an ergonomic standpoint, we subjectively observed that in the FANS group, fine stone fragments accumulated within the peri-scope annular space of the UAS, occasionally impeding smooth scope rotation and advancement. In contrast, the VISOR system’s intra-scope evacuation mechanism eliminated peri-scope fragment accumulation, and scope manipulation within the UAS remained unobstructed throughout all replicates. While this observation is qualitative and subject to operator bias, it suggests a potential ergonomic advantage of intra-scope suction designs that warrants objective quantification in future studies.

### Intrarenal pressure (IRP) safety

IRP control is a core safety priority in stone treatment, as multiple studies have confirmed that sustained IRP elevation above 30 mmHg increases the risk of pyelovenous backflow, bacterial translocation, and postoperative urosepsis, a life-threatening complication which can cause mortality [14, 32]. Although FANS can decrease IRP by means of external negative pressure aspiration, high IRP values can still be observed occasionally [25, 28].

The VISOR system’s distal-tip integrated pressure sensor provides millisecond-level real-time IRP monitoring, with automatic adjustment of irrigation and suction flow to maintain IRP within a relative low range. The system employs a two-tier IRP safety strategy: an audio-visual warning at 35 mmHg to enable proactive operator intervention, and mandatory automatic irrigation shutdown at 40 mmHg. In our study, the VISOR system maintained IRP below 40 mmHg throughout all procedures with a median IRP of 16.49 mmHg, well below the clinical safety limit in flexible ureteroscopy [33]. This proactive IRP control may reduce the risk of IRP-related complications, which would be particularly important for high-risk patients with infection stones or positive preoperative urine culture [34, 35]. However, this hypothesis requires clinical validation. The accuracy of the system IRP sensor performance was also validated in our study model through comparison with an external reference pressure meter (Spearman *R* = 0.99, *p* < 0.0001).

### Suction channel patency and fragment clearance dynamics

The VISOR system’s integrated suction-pressure monitoring provides real-time detection of working-channel occlusion via a two-tier safety design: an audio-visual alarm is triggered when suction circuit negative pressure reaches − 280 mmHg to alert the operator, and irrigation is automatically halted at − 350 mmHg to prevent further IRP escalation. This real-time pressure monitoring and built-in safety capability — although not fully challenged in this study due to the absence of blockage events — represents a potential safety feature for clinical practice.

The absence of suction channel blockage in this study reflects the combined effect of device design and fluid dynamics. First, the suction aperture mechanically restricts fragments larger than 1.8 mm from entering the channel. Second, the vortex flow—created by paired irrigation and suction—actively mobilizes fragments and retains them immediately in front of the scope tip. This allows for continuous laser fragmentation until the fragments are pulverized to a suitable size (≤ 1.8 mm) for smooth aspiration. Consequently, harder stone compositions or larger burdens will prolong laser lithotripsy time, but do not inherently increase the risk of channel occlusion.

### Additional clinical advantages

The VISOR system may also offer practical clinical advantages beyond pressure control and operative efficiency. Its integrated design reduces reliance on multiple separate devices, including an irrigation pump, negative-pressure suction device, and pressure-monitoring equipment. One potential advantage of this integrated system is that it may reduce the need for additional accessories that must be compatible with each other, thus streamlining surgical preparation and reducing the setup burden for operating room staff.

Compared with non-suction ureteroscopy, the VISOR system may also reduce the need for repeated stone basket retrieval, thereby minimizing intraoperative instrument exchanges and lowering consumable costs.

### Study limitations

Several limitations should be acknowledged. First, the primary limitation of this study is the small sample size (*n* = 3 per group), which precludes definitive statistical conclusions and limits generalizability. The high reproducibility observed across replicates — reflecting the standardized in vitro model with identical stone mass, operator, equipment, and laser parameters — supports the validity of the primary comparisons; however, larger-scale in vitro and subsequent clinical studies are required to confirm these findings. Second, this was an in vitro study performed using a 3D-printed static renal model, which does not reproduce in vivo renal perfusion or ureteral peristalsis. Moreover, all stone material in this study was placed in the renal pelvis, which masks the geometric challenges of lower-pole or multi-calyceal stones. Although the VISOR scope provides a bidirectional deflection angle of 270° to facilitate lower-pole access, its larger 12 Fr outer caliber may restrict entry into calyces with narrow calyceal necks. Dedicated clinical studies are required to evaluate calyceal access and stone clearance in these anatomically demanding configurations. Third, only calcium phosphate-predominant stones with a fixed mass of 3 g were evaluated. Performance with harder stone compositions (e.g. calcium oxalate monohydrate) or larger stone burdens, which may generate fragments more prone to channel occlusion, has not been assessed. Fourth, the two systems operated at different irrigation flow rates (VISOR: 155 mL/min; FANS: 100 mL/min), reflecting each device’s operating parameters for routine clinical use. This difference may have influenced stone suspension dynamics and operative efficiency; future studies with matched inflow conditions would enable a more controlled comparison of individual device characteristics. Fifth, the VISOR system utilizes a 12 Fr dual-channel flexible ureteroscope, which is larger in caliber than conventional single-channel ureteroscopes. This may limit its immediate applicability in unstented patients with a narrow ureteral lumen; pre-stenting may be required in such cases to facilitate safe scope passage and to avoid ureteral injury. Sixth, the realistic possibility of device malfunction due to fragment clogging, sensor malfunction or irrigation pump failure may indirectly make the entire scope non-functional, which could negatively affect the benefit of having a fully integrated system. Seventh, a fixed laser protocol (1 J × 30 Hz) was applied for this trial to exclude variations in lithotripsy as a confounding factor, enabling direct comparison of the native evacuation performance of the two systems. We acknowledge that device-specific differences in permissible fragment size may support individualized lasing strategies in clinical practice, which warrants further investigation. Eighth, the FANS group operated at a Ratio of endoscope-to-sheath diameter (RESD) of 0.625. As demonstrated in previous studies [36], an RESD < 0.85 is recommended when utilizing negative-pressure UAS. Further in vitro and in vivo studies are required to optimize RESD combinations for FANS-type systems. Ninth, the two groups differed inherently in scope caliber, irrigation flow rates, and automated IRP regulation. Because this study was designed as a pragmatic systems-level comparison rather than a single-variable bench test, these combined differences likely contributed to the shorter operative time observed in the VISOR group. Future studies with matched individual parameters are needed to isolate the contribution of specific design features. Additionally, the two-tier suction safety system for blockage detection was not challenged during this study due to the absence of obstruction events; its clinical sensitivity and reliability remain to be validated. Finally, irrigation fluid input-output balance was not systematically recorded in this study. Inclusion of comprehensive fluid dynamics data — including total irrigation volume, net fluid retention, and effluent fragment content — in future in vitro and clinical studies would provide a more complete characterization of each system’s hydrodynamic performance and intraoperative fluid management implications. Ultimately, this study assessed only immediate in vitro parameters; the long-term clinical safety and effectiveness of the VISOR system require confirmation by large-scale prospective clinical trials.

### Summary of key findings

In this standardized in vitro study, we conducted the first comparison between the novel VISOR integrated system and the mainstream FANS technique for stone treatment. Our key findings were as follows: (1) The VISOR system achieved a SCE comparable to that of FANS; (2) the VISOR system significantly shortened operative time by 26.2% compared with FANS, with similar total laser energy use; (3) The in-built IRP sensor of the VISOR system exhibited favorable measurement accuracy, along with consistent and stable intrarenal pressure control. If these can be replicated in real-world practice, these findings would support the further development of an all-in-one suction device category.

## Conclusion

Based on these preliminary in vitro findings, the VISOR integrated dual-channel system demonstrated encouraging results, achieving a SCE comparable to FANS, with significantly shorter operative time and reliable real-time IRP control. These results suggest that the VISOR system represents a promising candidate for further clinical evaluation; whether these performance advantages translate to improved patient outcomes in vivo requires confirmation in prospective clinical studies.

## Supplementary Information

Below is the link to the electronic supplementary material.

Supplementary Material 1

Supplementary Material 2

## Data Availability

The data used in the study to support the findings are available from the corresponding authors upon reasonable request.
